# β-Lactam Resistance and *Enterobacteriaceae*, United States

**DOI:** 10.3201/eid1109.050182

**Published:** 2005-09

**Authors:** Jean M. Whichard, Kevin Joyce, Paul D. Fey, Jennifer M. Nelson, Frederick J. Angulo, Timothy J. Barrett

**Affiliations:** * Centers for Disease Control and Prevention, Atlanta, Georgia, USA;; † University of Nebraska Medical Center, Omaha, Nebraska, USA

**Keywords:** *Salmonella*, *Escherichia coli* O157:H7, drug resistance, microbial, beta-lactamases, dispatch

## Abstract

Extended-spectrum cephalosporins (ESC) are an important drug class for treating severe *Salmonella* infections. We screened the human collection from the National Antimicrobial Resistance Monitoring System 2000 for ESC resistance mechanisms. Of non-Typhi *Salmonella* tested, 3.2% (44/1,378) contained *bla*_CMY_ genes. Novel findings included *bla*_CMY_-positive *Escherichia coli* O157:H7 and a *bla*_SHV_-positive *Salmonella* isolate. CMY-positive isolates showed a ceftriaxone MIC >2 µg/mL.

Extended-spectrum cephalosporins (ESC) are important for treating persons with severe *Salmonella* infections (*1*). This drug class is particularly important for pediatric therapy because fluoroquinolones are not approved for use in children. In 2000, 25% (8,153/32,022) of laboratory-confirmed *Salmonella* cases reported to the Centers for Disease Control and Prevention (CDC) occurred in children <5 years of age (*2*).

The National Antimicrobial Resistance Monitoring System (NARMS) for Enteric Bacteria began monitoring for resistance to cephalosporins and other drugs among human-derived *Salmonella* and *Escherichia coli* O157 isolates in 1996. *Shigella* was added to the surveillance in 1999. From 1996 to 1998, 15 (0.4%) of 4,093 non-typhi *Salmonella* isolates tested by NARMS were resistant to ESC, and none of the 675 *E*. *coli* O157 isolates tested were ESC resistant (*3*). Thirteen (87%) of 15 ESC-resistant *Salmonella* isolates exhibited a *bla*_CMY-2_-mediated mechanism of resistance (*3**,**4*): 11 serotype Typhimurium, 1 Thompson, and 1 Newport. One *S*. Cubana isolate exhibited a *bla*_KPC-2_ carbapenemase (*5*), and the remaining *S*. Typhimurium isolate exhibited a yet-uncharacterized extended-spectrum β-lactamase (*3*). To determine the dynamics and mechanisms of cephalosporin resistance among species and serotypes, we examined the 2000 NARMS collection and determined mechanisms of ESC resistance in isolates exhibiting elevated cephalosporin MICs.

## The Study

As NARMS participants, 17 state and local public health laboratories representing 40% of the US population submitted every tenth non-Typhi *Salmonella* isolate, every tenth *Shigella* isolate, and every fifth *E*. *coli* O157 isolate received in 2000 to CDC for antimicrobial susceptibility testing. Identification and serotyping were conducted at submitting laboratories. The MIC was determined for 17 antimicrobial agents at CDC by using partial range broth microdilution (Sensititre; Westlake, OH, USA). Isolates were chosen for further study based on the following MIC criteria: cefoxitin (>16 μg/mL), ceftiofur (>4 μg/mL), or ceftriaxone (>16 μg/mL).

### Isoelectric Focusing (IEF) for β-Lactamases

β-Lactamase content was determined for all isolates that met the MIC criteria for further study. The IEF methods of Rasheed et al. (*6*) were used with modification. Crude cellular protein extracts were prepared by pelleting 3-hour trypticase soy broth cultures (grown at 37°C with shaking at 300 rpm), resuspending in 0.2% sodium acetate at 5% of original culture volume, and freeze-thawing 4 times in a dry ice/ethanol bath and a 37°C water bath. Preparations were then diluted twofold with distilled water and placed on ice for 30 min with occasional swirling. The supernatant was collected after centrifuging for 30 min at maximum relative centrifugal force (14,000 rpm) in a Beckman 5417R microcentrifuge (Palo Alto, CA, USA). Three- to 5-µL aliquots of each preparation were resolved by focusing for 1.5 h on an Ampholine PAGplate polyacrylamide gel, pH range 3.5–9.5 (APBiotech, Piscataway, NJ, USA), according to manufacturer's instructions. Gels were stained by overlaying with a 500 µg/mL solution of nitrocefin (Becton Dickinson, Franklin Lakes, NJ, USA). Isoelectric points were estimated by comparison with the following standard β-lactamases: TEM-12 (pI 5.25), SHV-3 (pI 7.0) and MIR-1 (pI 8.4).

### Polymerase Chain Reaction (PCR) for β-Lactamase Genes

For isolates that were IEF-positive for a β-lactamase with a pI >8.4, amplification of *bla*_CMY_ genes was attempted. Internal primers were used to amplify a 369-bp portion of *bla*_CMY_ genes from crude colony lysates. The forward primer anneals to nucleotide (nt) 271–289 of the 1,146-nt *bla*_CMY-2_ sequence from *Klebsiella pneumoniae* (NCBI accession no. X91840) and has a sequence of 5´-GGCGTGTTGGGCGGCGATG-3´. The reverse primer anneals to nt 621-639 of *bla*_CMY-2_ and has a sequence of 5´-CAGCGGAACCGTAATCCAG-3´. APBiotech Ready-to-Go Beads (Piscataway, NJ, USA) were used to formulate 25-μL reactions, which were run in an MJResearch thermocycler (Waltham, MA, USA) under the following conditions: 1 cycle of 94°C for 5 min followed by 25 cycles of: 94°C for 30 s, 59°C for 1 min, 72°C for 1 min. Amplicons were resolved by electrophoresis in 1% agarose gels. For isolates exhibiting a β-lactamase with a pI = 8.0, *bla*_SHV_ genes were amplified using primers 4 and 5 from Rasheed et al. (*7*) with Perkin-Elmer Amplitaq Gold 2X Master Mix (Boston, MA, USA).

## Conclusions

In 2000, 2,152 non-Typhi *Salmonella*, *Shigella*, and *E*. *coli* O157 isolates were received and tested. Of these, 57 (2.6%) met the MIC criteria for additional testing to determine mechanisms of extended-spectrum cephalosporin resistance: 46 non-Typhi *Salmonella* isolates, 7 *Shigella* isolates (all *S*. *sonnei*), and 4 *E*. *coli* O157:H7 (Table). *bla*_CMY_ genes were identified in 44 (96%) of the 46 *Salmonella* isolates. One *S*. Nienstedten isolate produced a *bla*_SHV_ enzyme with a pI of 8.0. One *S*. Muenchen isolate with a cefoxitin MIC = 16 µg/mL yielded no β-lactamases by IEF. This isolate exhibited very low MIC of ceftriaxone and ceftiofur (<0.25 and <0.5 μg/mL, respectively).

**Table:** Characterization of isolates exhibiting increased MIC to extended-spectrum β-lactams among 2000 NARMS*

Isolate/serotype	Total no. met MIC criteria/total no. tested (%)	Total PCR-positive for *bla*_CMY_ (%)	Total IEF-positive for pl>8.4 (%)	No. that produce other β-lactamases
*Salmonella*				
Newport	27/124 (22)	27 (100)	27 (100)	1
Typhimurium	11/303 (3.6)	11 (100)	11 (100)	2
Heidelberg	3/79 (3.8)	3 (100)	3 (100)	1
Other nontyphoidal *Salmonella* (Agona, Infantis, Muenchen, Nienstedten, Reading)	5/872 (0.57)	3 (60)	3 (60)	1
*Shigella sonnei*	7/367 (1.9)	0	7 (100)	3
*Escherichia coli* O157:H7	4/407 (0.98)	4 (100)	4 (100)	0
Total	57/2,152 (2.6)	48 (84)	55 (96)	8

*NARMS, National Antimicrobial Resistance Monitoring System; PCR, polymerase chain reaction; IEF, isoelectric focusing.

The 7 *S*. *sonnei* isolates included in the study met only the cefoxitin MIC criterion (>16 µg/mL). All 7 showed a ceftiofur MIC 1.0 μg/mL or less, and a ceftriaxone MIC <0.25 μg/mL. Six of these were also resistant to ampicillin, amoxicillin-clavulanate, and cephalothin. Each isolate was IEF-positive for a β-lactamase enzyme with a pI>8.4, but was polymerase chain reaction–negative for a *bla*_CMY_ gene. We suspect the resistance is related to overproduction of chromosomal *amp*C genes known to be present in *Shigella* species (*8*); however, porin deficiency (*9*) and penicillin-binding protein changes (*10*) are worth exploration as well. Efflux mechanisms (*11**,**12*) are possible, but multidrug-resistance pumps might be less likely since none of the 7 isolates were resistant to chloramphenicol, nalidixic acid, or ciprofloxacin, and only 4 were resistant to tetracycline.

Eight isolates (5 *Salmonella* and 3 *S*. *sonnei*) produced putative TEM enzymes in addition to an enzyme with a pI>8.4. The pI in each case was 5.3 or 5.4. Plasmidborne *bla*_TEM-1_ enzymes have been identified in several *Salmonella* serotypes (*13*).

Twenty-seven (61%) of 44 *bla*_CMY_-containing *Salmonella* in 2000 were serotype Newport. This finding coincides with emergence of a multidrug-resistant strain of *S*. Newport called MDRAmpC (*14*). MDRAmpC increased from 1% (1/77) of *S*. Newport isolates tested by NARMS in 1998 to 22% (27/124) in 2000 (*15*). In addition, *bla*_CMY_ genes were found in 5 other *Salmonella* serotypes (Typhimurium, Heidelberg, Agona, Infantis, and Reading) in 2000. This contrasts with 1996–1998, when *bla*_CMY_ was found in 3 serotypes (Newport, Typhimurium, and Thompson), which indicated that these genes or the mobile elements that house them have been disseminated. Furthermore, *bla*_CMY_ genes were identified in each of the 4 *E*. *coli* O157:H7 isolates that met the MIC criteria in 2000. To our knowledge, this is the first report of *bla*_CMY_ in this *E*. *coli* serotype.

All 48 *bla*_CMY_-positive isolates (44 *Salmonella* and 4 *E*. *coli* O157:H7) exhibited a ceftiofur MIC >8 μg/mL; however, their ceftriaxone MIC ranged from 2 to 32 μg/mL. Since the Clinical and Laboratory Standards Institute (formerly National Committee for Clinical Laboratory Standards) breakpoint for ceftriaxone resistance is 64 μg/mL, none of these isolates were interpreted as ceftriaxone-resistant, and only 48% (23/48) were intermediate (16 or 32 μg/mL). In contrast, all 48 CMY-producing isolates showed a cefoxitin MIC >16 μg/mL (intermediate or resistant according to CLSI guidelines).

NARMS sampling in 2000 showed that *bla*_CMY_ genes continue to be the major mechanism of extended-spectrum cephalosporin resistance among non-Typhi *Salmonella*; other mechanisms of ESC are rare. The increasing diversity of *bla*_CMY_-positive *Salmonella* serotypes and the discovery of *bla*_CMY_ genes in *E*. *coli* O157:H7 highlight the mobility of these mechanisms. This finding is not unexpected since these genes have been shown to be present on large plasmids, some of which are transferable by conjugation (*4*). Since *S*. Newport MDRAmpC and *E*. *coli* O157:H7 have been associated with bovine reservoirs, we hypothesize that *bla*_CMY_ genes may be circulating among cattle. This remains to be proven and warrants more intensive study of *bla*_CMY_ prevalence and movement in bovine production settings. Further research is also necessary to determine factors that contribute to dissemination of the mobile elements carrying these genes and selection of *bla*_CMY_-positive strains such as *S*. Newport MDRAmpC. Notably, isolates exhibiting this extended-spectrum cephalosporinase may show a ceftriaxone MIC as low as 2 μg/mL, but MIC to ceftiofur and cefoxitin fall more reliably in the intermediate or resistant range. For this reason, we currently performed subsequent β-lactamase analysis on any isolate exhibiting a ceftriaxone or ceftiofur MIC >2 μg/mL.
